# Polymer Packaging through the Blending of Biowaste Oyster Shell and Low-Density Polyethylene: A Sustainable Approach for Enhanced Food Preservation

**DOI:** 10.3390/polym15193977

**Published:** 2023-10-03

**Authors:** Chang-Lei Qu, Shang-Ming Lin, Pranut Potiyaraj, Lei Meng, Chin-San Wu, Li Yuan, Xin Luo, Fei-Fan Ge, Chi-Hui Tsou

**Affiliations:** 1 School of Materials Science and Engineering, Sichuan University of Science and Engineering, Zigong 643000, China; 2 Material Corrosion and Protection Key Laboratory of Sichuan Province, Sichuan University of Science and Engineering, Zigong 643000, China; 3 Department of Materials and Textiles, Asia Eastern University of Science and Technology, New Taipei City 220, Taiwan; 4 Department of Petrochemistry and Polymer Science, Faculty of Science, Chulalongkorn University, Bangkok 10330, Thailand; 5 Department of Applied Cosmetology, Kao Yuan University, Kaohsiung 82101, Taiwan

**Keywords:** LDPE, oyster shell, tensile property, thermostability, blend film, antibacterial, barrier

## Abstract

This research delves into the impact of incorporating thermally treated oyster shell powder (TOS), a biowaste filler, into low-density polyethylene (LDPE) to develop a LDPE/TOS blend, aiming at enhancing food packaging materials. The LDPE/TOS blend portrays advantageous characteristics such as augmented mechanical strength, thermostability, crystallinity, water absorption, and improved hydrophobicity with TOS content up to 50%. Microstructure analysis reveals a transition from a sparse to a more interconnected structure, contributing to the amplified tensile strength. The blend demonstrates increased barrier properties against water vapor transmission, which is attributed to elongated diffusion paths induced by the TOS particles. Application of the blend material in vegetable preservation trials manifested a substantial reduction in water loss compared to pure LDPE or no packaging. This biowaste-based blend film extends the shelf-life of chicken significantly when compared to that of pure LDPE. Importantly, the LDPE/TOS blend exhibits excellent antibacterial properties against both *Escherichia coli* and *Staphylococcus aureus*.

## 1. Introduction

Polymer packaging materials are among the most important and commonly used applications in the current array of polymer usage scenarios. By 2019, the plastic packaging market was worth around $914.7 billion, indicating broad application prospects [1]. In recent years, with the rapid growth of the global population and increased levels of consumption, the demand for food packaging materials has continuously increased. Food packaging materials play a crucial role in the preservation, transportation, and sale of food [2]. As the food packaging industry evolves, the requirements for material performance, environmental friendliness, and sustainability are becoming increasingly stringent. Low-density polyethylene (LDPE), due to its ease of processing and molding, as well as its superior flexibility and sealability, has been widely used in polymer packaging materials [3]. However, traditional food packaging materials, such as polyethylene (PE), while possessing good mechanical and processing properties, lack robust antibacterial performance. This shortfall could lead to microbial contamination issues during food storage [4]. Therefore, the research and development of novel food packaging materials with antibacterial functions to meet the requirements of food safety and public health have become increasingly important.

Reesha et al. utilized maleic anhydride grafted LDPE as a compatibilizer. They uniformly incorporated chitosan at concentrations of 1%, 3%, and 5% (*w/w*) into the LDPE matrix, yielding an innovative antimicrobial packaging material. The resulting material was tested as a packaging medium for the refrigerated preservation of tilapia. Upon evaluation, the preservation efficacy of this packaging was found to be superior. The newly developed chitosan-integrated blend film demonstrated exceptional antimicrobial characteristics, thereby significantly prolonging the shelf-life of the tilapia [5]. LDPE film was endowed with antimicrobial capabilities using copper hydroxy nitrate. The blend material, loaded with 0.3% CuHS, exhibited lower cytotoxicity, thereby attesting to its relative safety [6]. LDPE was enriched with molybdenum disulfide (MoS_2_) nanoparticles at varying concentrations of 0.5%, 1%, and 3%, leading to the development of antimicrobial blend packaging materials. Although these materials demonstrated superior antibacterial properties against *Salmonella typhi* and *Listeria monocytogenes*, which could be attributed to both the photoactivity of the filler and changes on the blend surface, a diminished barrier performance was observed [7]. Nanoblends were prepared using a wet casting method, incorporating a hydrothermally synthesized lithium-titanate (Li-TiO_2_) photocatalyst and subsequently combining it with LDPE. This innovative material exhibited excellent antibacterial properties, proving effective in the inactivation of *Staphylococcus aureus*. The introduction of lithium ions adjusted the band gap of TiO_2_, thereby enhancing its responsiveness to visible light, reducing electron hole recombination, and improving cell inactivation efficiency. The blend material, containing 1 wt% Li-TiO_2_, was capable of inactivating 99% of *Staphylococcus aureus* within a 12 h timeframe [8].

Nano-TiO_2_/LDPE packaging materials were fabricated through the utilization of a twin-screw extruder, effectively enhancing the barrier performance of the blend materials. In specific strawberry fruit tests, it was found that when the TiO_2_ addition was at 1%, there was maximum protection of the strawberry’s flavor and nutritional quality [9]. LDPE/CaCO_3_ packaging material, which significantly inhibited the browning of fresh-cut yam slices under 10 °C storage conditions, demonstrating good preservation effects [10]. However, to protect food from bacterial intrusion and extend the shelf-life, polymer packaging materials need to have strong antibacterial properties. Blend film materials were developed employing low-density polyethylene (LDPE), organically modified montmorillonite clay (MMT), and cinnamaldehyde. Compared to simple LDPE/cinnamaldehyde, the addition of MMT-modified blend films exhibited more durable antibacterial activity against *Escherichia coli* (*E. coli*) and *harmless Listeria*. Additionally, adding a small amount of metal components to polymers is a common method for imparting antibacterial properties to blend materials. Typically, metals induce an electric field on the microbial film, altering the permeability of microbial cells, or directly affecting the microbial enzyme system and bacterial metabolism, achieving the effect of killing bacteria [11]. Blend materials were prepared by incorporating copper-modified montmorillonite (MtCu_2_^+^) into blended LDPE. The study showed that when the filler was at 4%, the *E. coli* colonies decreased by 94%, effectively inhibiting the activity of *E. coli* [12].

In summary, most antibacterial fillers currently used are polysaccharides, metal ions, or nanomaterials, all of which come with high costs. If antibacterial fillers sourced from biological waste can be used, it would substantially reduce the cost of food packaging materials. The utilization of calcined oyster shell powder (TOS) as an antibacterial filler for polypropylene (PP) was initially introduced in the literature. The results indicated that at a 0.5% addition, it not only possessed good antibacterial activity but also improved the mechanical strength of PP [13]. Another study also employed TOS as a filler for polyphenylene sulfide (PPS) to produce PPS/TOS blend materials. The results showed that TOS could not only endow PPS with antibacterial activity against *Staphylococcus aureus* (S. *aureus*) and *E. coli*, but could also enhance the tensile strength, bending resistance, thermal decomposition temperature, and fatigue strength of PPS [14]. LDPE exhibits notably inadequate oxygen barrier characteristics [15], making it unsuitable for packaging applications that require long-term oxygen barrier performance. While LDPE lacks the oxygen barrier capabilities of other polyolefins like polypropylene and high-density polyethylene, as well as other polymers like polyamide and polyethylene terephthalate [16], it offers other advantageous properties such as flexibility, toughness, transparency, sealability, and ease of opening. These attributes make it suitable for the short-term preservation of easily observable fresh foods. Therefore, the aim of this study is to evaluate the preservation efficacy of LDPE in short-term food packaging applications, specifically for vegetables and meats. This study augmented treated oyster shell waste as a filler for LDPE, which can not only significantly reduce the cost of the blend material, but can also enhance its mechanical performance, thermal performance, and barrier properties, and give it excellent antibacterial activity. Special attention will be given to the blend’s effectiveness in extending the shelf-life of Chinese cabbage and chicken. The study will culminate in the identification of the optimal filler concentration, thereby providing a comprehensive assessment of the material’s potential for food packaging applications.

## 2. Experimental

### 2.1. Materials

LDPE (2426 H, Mw = 30,000) with a milky white appearance and density of 0.924/cm^3^, was obtained from China Petroleum and Chemical Corporation Maoming Branch, Maoming, China). Oyster shells were collected from the coast of Fujian Province, China, and ground to 2000 mesh via ball mill from Wuxi Xinerli Machinery Equipment Co., Ltd. (XLM-2, Wuxi, China) The shells were then subjected to high-temperature calcination in a muffle furnace from Shanghai Shijie Electric Furnace Co., Ltd. (SG-XS, 1700 °C, Shanghai, China) to produce thermally treated oyster shell powder (TOS). Specific calcination conditions were as follows: temperature at 900 °C, holding time of 1 h, an atmosphere of air, and a heating rate of 10 °C/min.

### 2.2. Preparation of LDPE/TOS Blends

**Figure 1:**
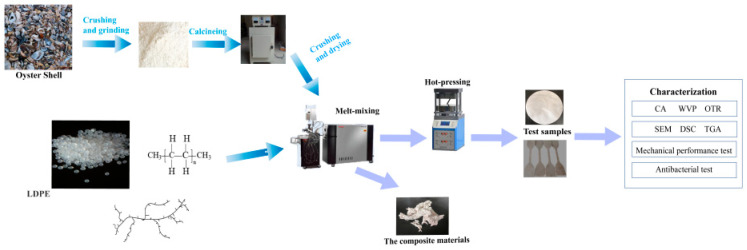
Diagram for processing LDPE and LDPE/TOS blends.

Figure 1 shows the process of preparing LDPE and LDPE/TOS blends. (1) Material drying: LDPE was dried in an oven at 85 °C for 8 h until completely dry. The dried material was then kept in a constant-temperature drying oven at 60 °C. TOS was dried in an oven at 105 °C for 4 h, then at 85 °C for 8 h until completely dry. (2) Preparation of LDPE/TOS Blends: The LDPE and two different particle sizes of TOS were melt-blended in a certain ratio (Table 1) using a torque rheometer (HAAKE PolyLab OS, Type567–0021, Germany). The experiment was conducted at 160 °C, with a rotation speed of 80 rpm/min for the first 3 min. The speed was then adjusted to 150 rpm/min for the next 2 min to produce the LDPE/TOS blends. These blends were hot pressed at 160 °C for 10 min using a vulcanizer. The material was then removed from the vulcanizer for cooling. A custom cutter was used to cut the material into dumbbell-shaped samples, which were then used for testing and characterization. The components and their amounts for each sample are shown in Table 1.

### 2.3. Mechanical Properties

The tensile strength and elongation at break of the samples were measured using a universal tensile testing machine (ASTM D638 type, Shenzhen, China). A fixed crosshead speed of 10 mm/min was employed, with five parallel measurements taken and averaged for the final result [17].

### 2.4. Morphology

Samples were attached to a sample support, then sputtered with a layer of gold. Through scanning electron microscopy (SEM, HITACHI, S-3400N, Tokyo, Japan), operated at an accelerated voltage of 20 kV, the cross-sectional images of LDPE/TOS blends were captured [18].

### 2.5. Thermogravimetric Analysis (TGA)

First, 8–10 mg of samples was weighed. After that, drying of the samples was done at 105 °C for 4 h [19]. Then, thermogravimetric analysis (TGA) (HTG–1, Beijing Hengjiu Experimental Equipment, Beijing, China) was used to test for the thermal stability under nitrogen atmosphere. During the testing, the temperature was kept at 25 °C for 30 min, and then increased to 750 °C at a constant rate of 10 °C/min [20]. Generate a temperature-weight loss ratio graph using Origin software (Version 2010) with the obtained test data. Additionally, include the initial degradation temperature and the temperature of maximum degradation value on the graph.

### 2.6. Differential Scanning Calorimetry (DSC)

With the use of a TA Q100 DSC instrument, the samples were subjected to heating to determine the thermal properties. Heating was conducted at a rate of 10 °C/min, while the samples under flowing nitrogen at a flow rate of 50 mL/min were scanned [21]. $X_{c}$ or the degree of crystallinity was calculated using Equation (1):

(1) $$X_{c}=\frac{\Delta H_{m}}{wt\%\times \Delta H_{m}^{0}}\times 100\%\;$$

wt% = percentage of LDPE in of LDPE/graphite blends, %;

∆*H_m_* = measured enthalpy of fusion, J/g;

∆*H_m_*^0^ = theoretical enthalpy of LDPE at 100% crystallization, 293 J/g.

### 2.7. Contact Angle

The hydrophilicity of the nanoblends was assessed using a contact angle tester (JC2000D, Shanghai Zhongchen Digital Technology Equipment Co., Ltd., Shanghai, China). The contact angle serves as an indicator of a solid surface’s wettability. Smaller test results signify better sample wettability, while larger results indicate lower wettability. A micro syringe was employed to extract 2 μL of distilled water, which was then dropped onto a sample, and contact angles were recorded using a five-point fitting method. All samples were measured in triplicate to obtain an average value. Test parameter settings included a film thickness of 1 mm, a test duration of 3 s, and three test measurements [22].

### 2.8. Water Uptake Rate

The water uptake (WU) rate was assessed following standard methods. LDPE/TOS samples were cut into 2 cm × 2 cm pieces and dried to a constant weight in an oven at 100 °C. Prior to the water absorption test, the samples were weighed and immersed in distilled water at room temperature for 24 h [23,24]. Afterward, they were removed from the water, gently patted dry with a tissue paper to remove water droplets, and weighed again. Water absorption was calculated using Equation (2), where WU denotes water absorption (g/g), mo represents the sample weight (g) before soaking in distilled water, and mf indicates the sample weight (g) after soaking.

(2) $$\mathrm{WU}\;(g/g)\;=\frac{m_{f}+m_{O}}{m_{O}}\times 100\%\;$$

### 2.9. Water Vapor Permeability

On the basis of ASTM E398-13 [25,26]. A water vapor transmission rate test system (W3/060, Jinan Languang Electromechanical Technology Co., Ltd., Jinan, China) was employed to evaluate the water vapor barrier properties of LDPE/TOS materials, following the ASTME96 standard. Prior to testing, samples were prepared as circular films with a diameter of 75 mm and a thickness of 0.1–0.2 mm. The test chamber temperature was set at 25 °C, with a test time interval of 30 min and a humidity level of 89% within the chamber.

The water vapor transmission coefficient was used to compare the test results and standard values of various samples with differing thicknesses. The findings in this study were expressed in terms of water vapor permeability (WVP), using the unit g·cm/cm²·s·Pa. The WVP calculation formula is as follows:

(3) $$P_{v}=\frac{\Delta m\cdot d}{A\cdot t\cdot \Delta P}$$

where Pv = water vapor permeability, g·cm/cm^2^·s·Pa;

∆m = mass increment within a certain time t, g;

t = time interval after the mass increment stabilization, s;

d = sample thickness, cm;

A = area of sample available for water vapor permeation, cm^2^;

∆P = water vapor pressure difference across the sample, Pa.

### 2.10. Vegetable Preservation Test

Fresh Chinese cabbage was purchased from the market for the assessment of preservation state and rate of water loss (RWL). The surface moisture of the fresh vegetables was wiped with dry tissue paper and the initial weight (*W*_0_) of the vegetables was recorded. The blend material was then rolled into a film to wrap the wiped vegetables, with unwrapped vegetables serving as the control group. After being left at room conditions (25 °C and 70% RH) for four days, the vegetables were weighed again (*W_dry_*). The RWL was calculated using the following formula:

RWL = (*W*_0_ − *W_dry_*)/*W*_0_ (4)

RWL = rate of water loss

*W*_0_ = initial weight

*W_dry_* = the weight at room conditions (25 °C and 70% RH) for four days

### 2.11. Antibacterial Test

(1) Qualitative antibacterial test: The prepared LDPE/TOS blend was vulcanized and pressed into a film, then cut into 1 cm circular films for further use. Solid media were prepared by weighing 10 g of peptone, 5 g of yeast powder, 10g of NaCl, 25 g of agar, then adding 1000 mL of hot deionized water, stirring well until the agar fully dissolved. Afterward, the conical flask mouth was wrapped with a test paper, sealed with thin rope, and sterilized in a high-pressure steam sterilizer at 120 °C for 20 min. The sterilized flask and culture dish were exposed to ultraviolet light for 30 min, then cooled to around 40 °C. Culture media were then poured under a super clean bench and left to solidify for further use. Bacterial inoculation: A pipette was used to drop 0.1 mL of active *E. coli* onto the solid media, and it was spread using the spread plate method to ensure an even distribution on the surface of the solid media. A small amount of sterilized TOS was poured into the center of the solid media, covered, and inverted in an incubator at 37 °C. After 24 h, the size of the inhibition zone was observed [27].

(2) Quantitative antibacterial test: Round films of each blend were prepared first [28,29]. Liquid media were prepared using the same components as the solid media, excluding the agar, and distributed into seven conical flasks. Each flask was inserted with a sample, inoculated with a small amount of bacteria, shaken well, labeled, and placed into a shaking incubator (BS-1E, Changzhou Jintan Liangyou Instrument Co., Ltd., Changzhou, China) for 12 h. The bacterial solutions from each conical flask were diluted quantitatively. For instance, for pure LDPE bacterial solution, prepare 5 long test tubes, labeled as 10, 10^2^~10^5^, each filled with 9 mL of sterile water. A sterile pipette was used to take 1 mL of pure LDPE bacterial solution, which was then added to the test tube labeled ‘10’ and shaken to mix. The process was repeated, with 1 mL taken from the ‘10’ test tube and added to the ‘10^2^’ test tube, shaken to mix, and so on until the ‘10^5^’ test tube was reached, achieving a 10^5^ times dilution. Then, 0.1 mL was taken from the last diluted solution and spread onto the solid media, using the spread plate method for even distribution. After covering and incubating for 12 h, the bacterial colonies were counted.

### 2.12. Chicken Preservation Test

Fresh chicken, slaughtered on the same day of purchase, was obtained from the market for the preservation and antibacterial tests using the blend material. During the transportation of the sample, ice cubes were placed inside the insulation bag. The chicken breast was sliced into circular pieces, each weighing 1 g. The blend material, with dimensions of 80 mm in length, 80 mm in width, and 0.3 mm in thickness, was subsequently formed into a film for enveloping the chicken pieces. Unwrapped chicken pieces were employed as the control group for comparison. The samples were then stored under normal refrigerator conditions (4 °C). After the preservation period, the wrapping film was opened, and the chicken pieces were taken out and placed in conical flasks containing 50 mL of physiological saline solution. The content was shaken until turbid, and a tenfold dilution method was employed for bacterial counting. The specific counting procedure involved preparing small centrifuge tubes labeled as 10, 10^2^, 10^3^ to 10^8^. Each tube was filled with 9 mL of physiological saline solution. The turbid culture liquid in the conical flask was subjected to tenfold dilution. Using a sterile pipette, 1 mL of the diluted conical culture liquid was drawn and added to the tube labeled 10, followed by shaking for uniformity. The pipette was then replaced, and 1 mL of the diluted solution from the tube labeled 10 was transferred to the tube labeled 10^2^, again with shaking. This process of dilution was repeated stepwise. Finally, 0.1 mL of the last diluted solution was taken and gently spread onto a solid culture dish, ensuring thorough mixing of the medium with the bacterial solution. After covering, the dishes were incubated at a constant temperature for 24 h. Subsequently, bacterial counts were performed on samples containing 200–300 colonies (with three parallel repetitions for each dilution factor). The value obtained represented the colony-forming units (CFU). The concentration of bacteria on the surface of the chicken was measured at different time intervals: 0, 12, 24, 36, and 48 h.

### 2.13. Statistical Method

All experiments were conducted in quintuplicate (n = 5), and the test results were presented as mean values with standard deviations (Mean ± SD) [30]. A completely randomized design (CRD) was employed for all experiments, with each experimental treatment replicated three times. Finally, statistical analysis and graphical representation of all data were performed using Origin software (version 2021).

## 3. Results and Discussion

### 3.1. Tensile Strength

**Figure 2:**
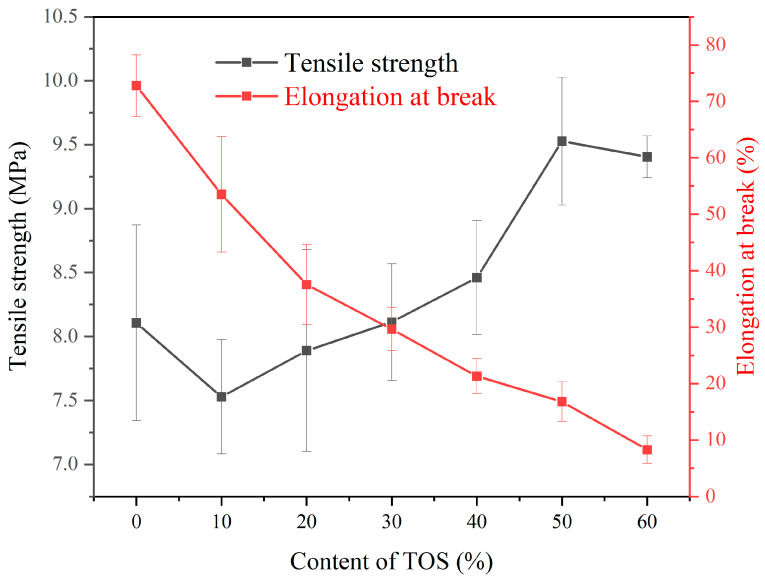
Stress and elongation at break of LDPE blends containing different amounts of TOS.

As observed in Figure 2, upon adding 10% TOS into the LDPE matrix, there was a slight reduction in tensile strength. Starting from 20% TOS, the tensile strength of the blend materials initially increased with the increment of TOS content. The maximum tensile strength, 9.5 MPa, was achieved when 50% TOS was incorporated, exhibiting an improvement of 17.3% compared to pure LDPE. When TOS content is low, the distance between filler particles is relatively large and the interaction with the matrix material is not sufficient to result in a significant improvement in mechanical properties, thus, the strength could decrease. However, as the content of TOS increases, the distance between filler particles decreases, forming an effective filler–matrix interface that can disperse load, thereby significantly enhancing the material strength. The presence of filler particles also impedes the slippage and plastic deformation of the matrix material, contributing to the increase in strength [31,32]. However, when the TOS content reached 60%, a decrease in tensile strength was observed, likely due to the agglomeration of TOS, leading to a decline in mechanical performance. As can be seen from Figure 1, the elongation at break of the LDPE/TOS blend materials steadily declined as the TOS content increased. This could be attributed to the rigidity of TOS filler particles, which possess a lower elongation at break compared to LDPE. As the TOS content increased, the distance between LDPE molecular chains enlarged, weakening the intermolecular forces and leading to a decrease in the elongation at break of the blend materials. While the overlapping error bars in the TOS tensile strength and elongation at break data may introduce a degree of measurement uncertainty, it is crucial to emphasize that even a marginal difference of 1 MPa can result in variations exceeding 10% in tensile strength, given the inherently modest strength of pure LDPE. Nonetheless, the observable ascending and descending trends in both tensile strength and elongation at the break offer compelling evidence supporting the reliability of the tensile performance test outcomes.

### 3.2. Morphology

**Figure 3:**
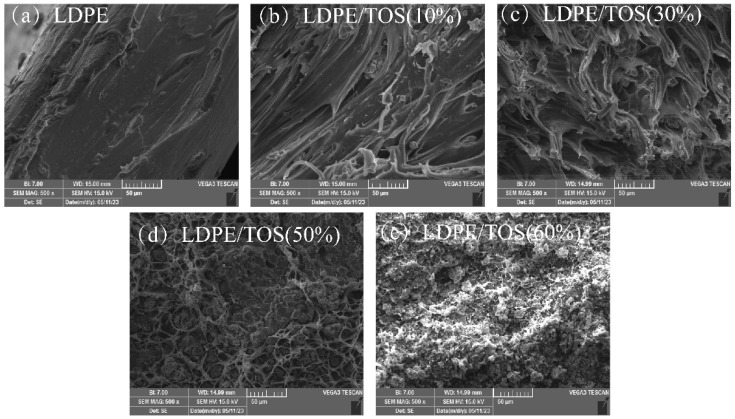
SEM micrographs of the fracture surface morphology of LDPE/TOS blends with various amounts of TOS.

The SEM images of the tensile fracture surface of the LDPE/TOS blend materials are shown in Figure 3. The microstructure of the LDPE/TOS blends is significantly influenced by different TOS additive levels. The surface of the pure LDPE sample is regular, continuous, uniform, and exhibits a unidirectional structure, typically characteristic of a ductile structure [33]. With an addition of 10% TOS, the blend material presents a relatively sparse unidirectional structure. Corresponding to the tensile property analysis, the mechanical strength is lowest at this concentration, possibly due to TOS disrupting the continuity of the LDPE. When the addition amount is less than or equal to 30%, the blend material still appears somewhat sparse, but it presents a network-like structure. This may be because TOS begins to gradually play a reinforcing role, hence the blend material with 30% TOS shows slightly increased strength compared to 10–20% TOS, but a significant decrease in ductility is observed. At a TOS addition of 50%, the morphology begins to clearly show TOS particles, presenting a more regular shape and closely connected network structure. This is due to the sufficient amount of TOS at this level, truly playing a reinforcing role in LDPE [34]. With a TOS content of 60%, the continuity of LDPE is disrupted, TOS cannot be uniformly dispersed in LDPE, severe agglomeration occurs, large voids appear, and the network structure is lost. As a result, a reversal in tensile strength is observed, and the elongation at break reaches the minimum value.

### 3.3. TGA

**Figure 4:**
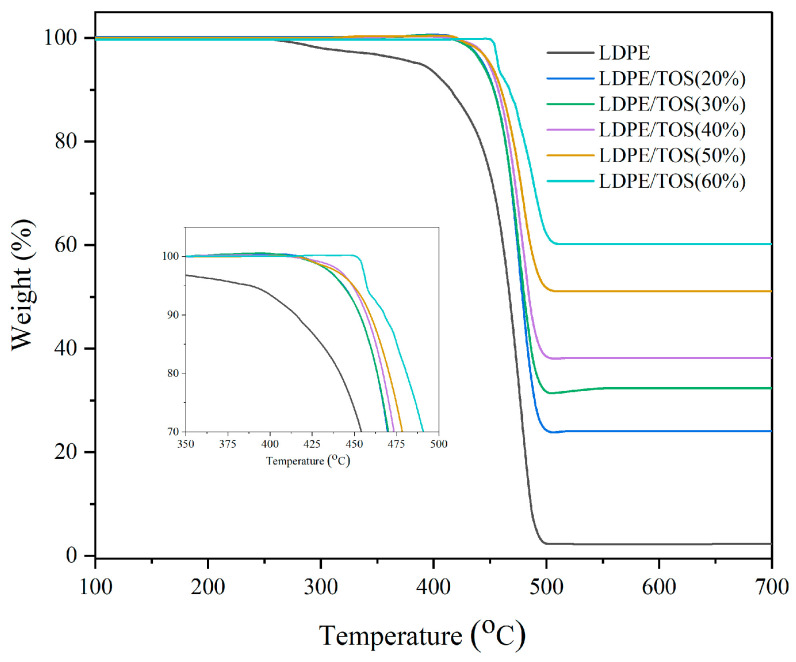
TGA of LDPE and LDPE/TOS blends.

Figure 4 and Table 2 display the TGA analysis curves and data for LDPE/TOS blends. From the graph and table, it is evident that as the TOS content increases, both the initial cracking temperature (*T_onset_*) and the maximum cracking temperature (*T_max_*) of LDPE exhibit an upward trend. This could be attributed to the fact that TOS itself is a filler that has been sintered at high temperatures, so essentially, it does not lose weight before 700 °C. Therefore, the high-temperature-resistant TOS can significantly enhance the thermal cracking temperature of LDPE [35], especially the initial cracking temperature. Moreover, TOS particles might act as barriers that hinder the permeation of volatile degradation products within the LDPE matrix. This barrier effect could delay the onset of thermal degradation, thereby elevating the degradation temperature [36].

### 3.4. DSC

**Figure 5:**
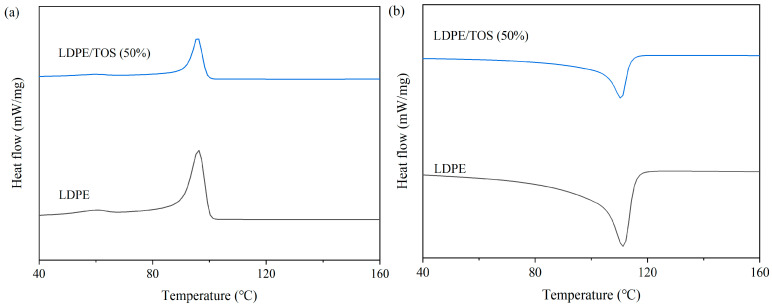
DSC curves of LDPE and LDPE/TOS blends: (**a**) cooling curve; (**b**) heating curves.

Figure 5 and Table 3 present the DSC curves and data for LDPE/TOS blends. From these, it can be observed that the glass transition temperature (*T_g_*), crystallinity (*X_c_*), and melting temperature (*T_m_*) of LDPE all show a slight increase when the TOS content is at 50%. The increase in *T_g_* may be attributed to the presence of TOS, which to some extent inhibits the mobility of the LDPE molecular chains [37]. This inhibition might promote the growth or increase the density of crystallinity within the polymer matrix, thereby enhancing the crystallinity of the blend material. The rise in *T_m_* could be due to several reasons: (1) TOS facilitates the crystallization of LDPE, and the increased crystalline regions may elevate the melting point of the blend material, as crystalline areas melt at higher temperatures than amorphous ones. (2) TOS might act as a physical barrier within the LDPE matrix, restricting the motion of polymer chains, possibly increasing the energy required for melting, and thereby raising both *T_m_* and *T_g_* [38,39]. (3) As indicated by the TGA analysis, the high thermal stability of TOS itself could contribute to enhancing the overall thermal stability of the blend material, including an increase in the melting point.

### 3.5. Water Absorption and Hydrophilicity

**Figure 6:**
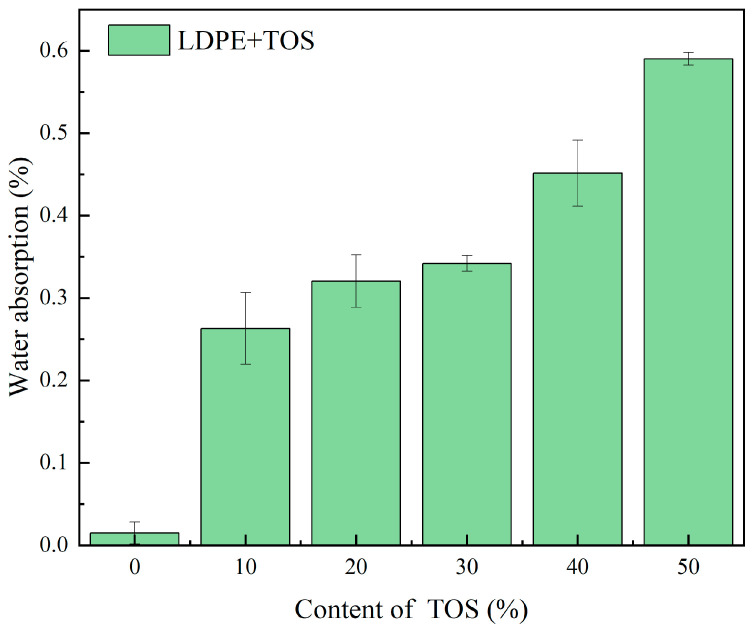
Water uptake for LDPE/TOS blends with different amounts of TOS.

**Figure 7:**
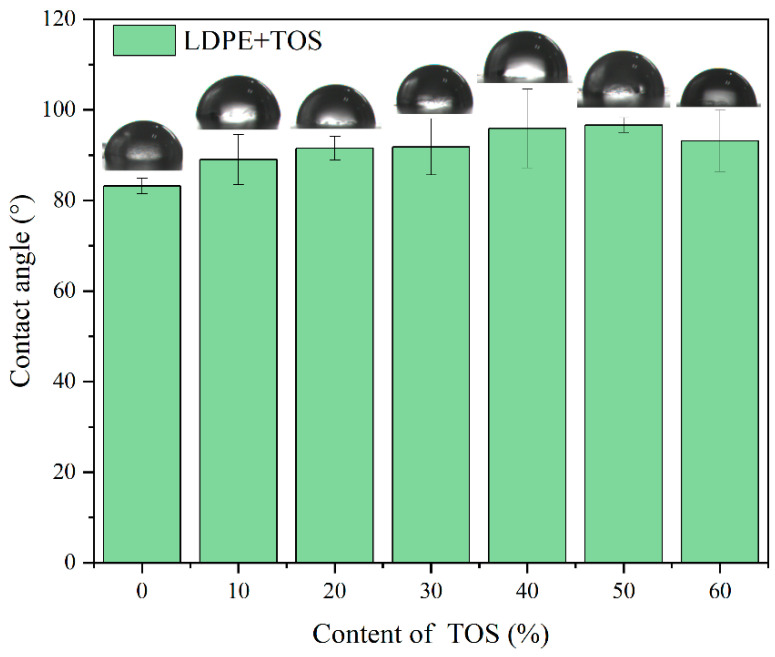
Contact angle images and values for LDPE/TOS blends with different amounts of TOS.

The water absorption rate and water contact angle test results for the LDPE/TOS blend materials are depicted in Figure 6 and Figure 7. In Figure 6, it can be observed that as the amount of TOS gradually increases, the water absorption rate of the blend material presents an upward trend, with all blend materials reaching a maximum value of 0.59% at a TOS content of 50%. This may be caused by two reasons. Firstly, the main component of calcined TOS is calcium oxide, which has hygroscopic properties [40], leading to an increase in the water absorption of the blend material. Secondly, there may be interface defects between TOS and LDPE, which would increase the space for water storage.

Figure 7 shows the water contact angle of the LDPE/TOS blend materials, specifically, the test results when a water droplet has been on the surface of the blend material for 3 s. The contact angle of the LDPE TOS blend material essentially presents a trend of first increasing and then decreasing. The contact angle of pure LDPE is 83.2°, and when the TOS additive amount reaches 50%, the contact angle value is at a maximum of 98.7°. This suggests that the hydrophobicity of the blend material surface increases as the TOS content increases. This may be due to two reasons. Firstly, the presence of TOS can increase the surface density, making it more difficult for water molecules to penetrate the blend material in a short time. Secondly, the presence of TOS might elevate the surface roughness of the blend material. LDPE is a non-polar hydrophobic material, and the larger the surface roughness, the larger its contact angle [29,41]. However, when the TOS content reaches 60%, the water contact angle decreases, possibly due to TOS agglomeration in the matrix, which results in the appearance of surface pores, leading to a decrease in contact angle.

### 3.6. Barrier Properties

**Figure 8:**
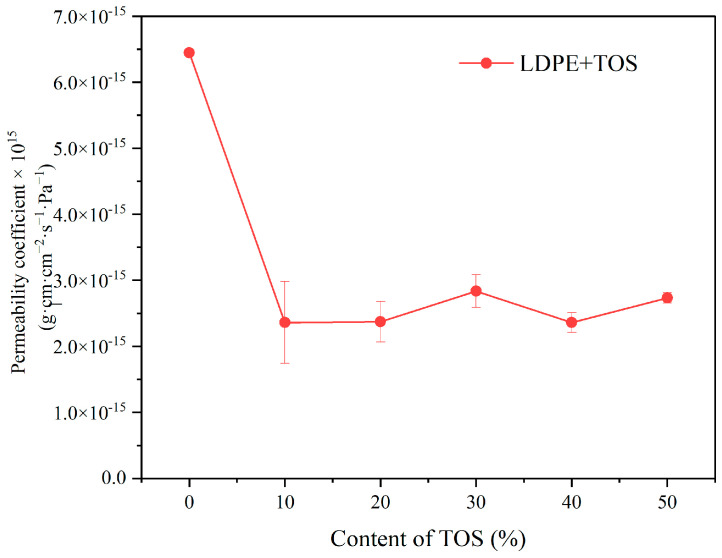
Water molecule barrier effect for LDPE and LDPE/TOS blends with different amounts of TOS.

Figure 8 presents the variation curve of the water vapor permeability coefficient of LDPE/TOS blend materials with different TOS contents. As can be seen from the figure, with the addition of 10% TOS, the water vapor permeability coefficient of the blend material is significantly lower than that of pure LDPE. This suggests that the addition of TOS can markedly enhance the barrier performance of LDPE. However, when the TOS content is further increased, its impact on performance is not significant, with all values appearing similar. Although the permeability coefficient is lowest when the TOS content is at 40%, at 2.362 × 10^−15^, it is not significantly different from the values obtained at other TOS contents.

**Figure 9:**
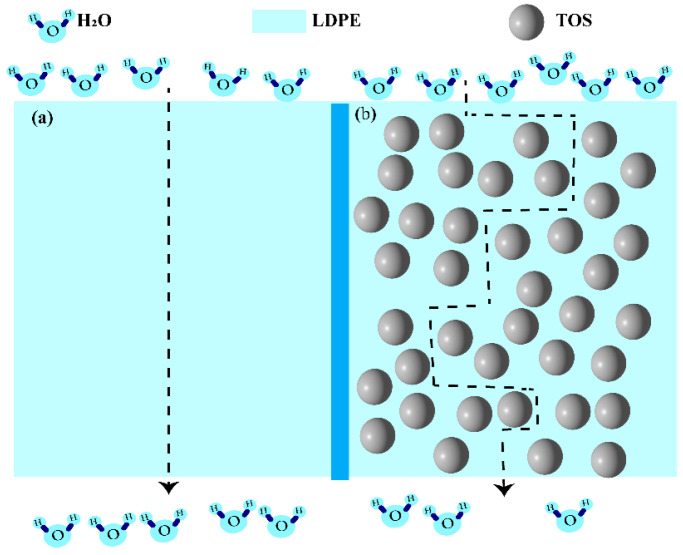
Schematic diagram of the barrier for (**a**) LDPE and (**b**) LDPE/TOS blends with different amounts of TOS.

The diffusion of water molecules in the blend material is illustrated in Figure 9. In pure LDPE, water molecules disperse along the vertical path (Figure 9a) [42,43]. In the TOS-containing blend material, due to the presence of large TOS particles, the molecules can only move around them (Figure 9b), which in turn extends the movement path of the water molecules [44,45,46] and enhances the barrier performance.

### 3.7. Vegetable Preservation Experiment

**Figure 10:**
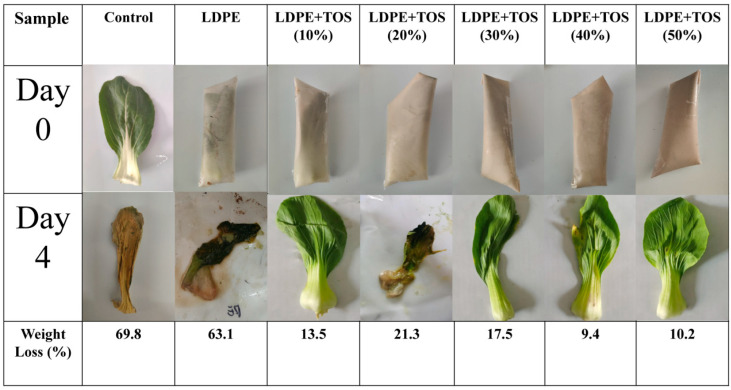
Water loss rate of vegetables.

Figure 10 presents the weight loss of vegetables, both packaged in blend material and unpackaged, after 4 days at room temperature. It can be observed that, at room temperature, the water loss in unpackaged vegetables is severe, with a moisture loss rate of 69.8%. Using pure LDPE material with poor barrier properties to wrap vegetables still results in poor preservation effects [47,48], as the vegetables undergo dehydration and decomposition, equivalent to the vegetables being directly exposed to the room temperature environment (Figure 10). After using LDPE/TOS blend material to wrap the vegetables, the weight loss rate of the vegetables significantly decreases in all instances. Comparatively, wrapping vegetables in blend material with a TOS filler content of 40% can noticeably reduce the weight loss rate of the vegetables, with a loss rate of 9.4%. This is likely due to TOS enhancing the barrier properties of LDPE. These results indicate that, compared to pure LDPE, the addition of TOS can extend the preservation of vegetables for a longer period.

### 3.8. Shelf-Life of Chicken Meat

**Figure 11:**
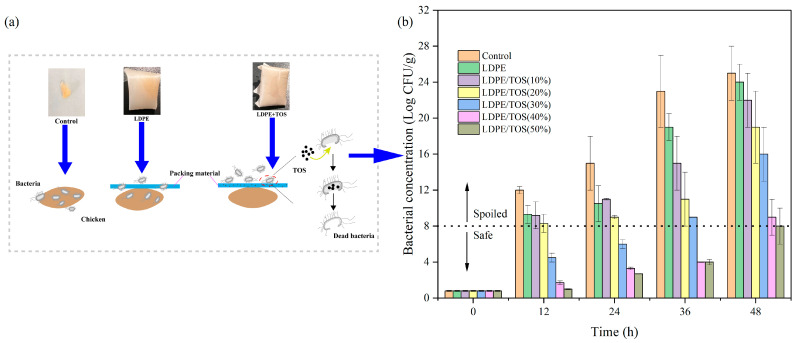
(**a**) Proposed antibacterial activity in raw chicken; (**b**) inhibitory effect of LDPE and LDPE/TOS blends with different amounts of TOS.

According to the research by Bolton et al. [49], the shelf-life of chicken meat chilled at 4 °C under aerobic conditions was less than 5 days. The shelf-life of fresh chicken stored in the refrigerator at 4 °C was about 3 days. Meat is at risk of spoilage when the bacterial count reaches 10^7–8^ CFU/g [2,49]. Typically, when chicken is stored in a refrigerator, bacteria within the fridge can penetrate the packaging film, contaminating and spoiling the chicken. However, the TOS in the LDPE/TOS blend can disrupt the bacterial cell wall, leading to bacterial death (Figure 11a). Consequently, the blend material can protect the chicken from bacterial contamination to a certain degree. In this study, we measured the bacterial count on the surface of the raw chicken every 12 h. It is evident that both the control group and the pure LDPE film have almost no inhibitory effect on bacterial growth. However, the addition of TOS to the blend material significantly enhances its antibacterial properties. Particularly, when the TOS content is ≥30%, the bacterial growth between 12–36 h is noticeably inhibited, with the blend material effectively controlling the bacterial count below the level that causes spoilage. After 36 h, the microbial growth rate accelerates significantly. Yet, the LDPE/40%TOS and LDPE/50%TOS blends continue to exhibit good antibacterial properties (Figure 11b). Chicken wrapped in blend material with a TOS content of 50% remains within safe levels even after 48 h. These results indicate that the LDPE/TOS blend can maintain the quality of fresh chicken for a certain period, with the antibacterial performance improving as the TOS content increases. Since first publicly stated that TOS can impart antibacterial properties to polymers [13], these results were expected, further confirming the feasibility of incorporating TOS into LDPE food packaging materials.

### 3.9. Antibacterial Test

**Figure 12:**
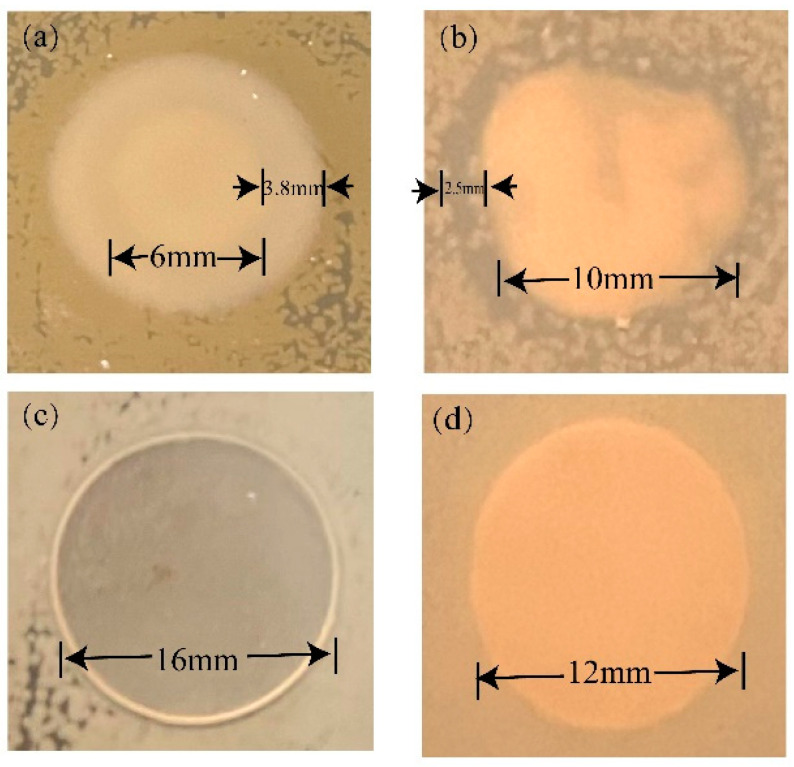
Qualitative analysis of antibacterial activity: (**a**) TOS for *E. coli* and (**b**) TOS for *S. aureus*; (**c**) LDPE/TOS(50%) blends for *E. coli;* (**d**) LDPE/TOS(50%) blends for *S. aureus*.

Figure 12a,b illustrated the inhibitory effect of TOS on *E. coli* and *S. aureus* colonies, displaying a significant antibacterial zone. However, when TOS was incorporated with LDPE to form a blend, no prominent antibacterial zone could be observed in the blend antibacterial assay. Figure 12c,d depicted the performance of the blend material with a 50% TOS addition within *E. coli* colonies and *S. aureus*, where no distinct inhibitory zone was observed. This necessitated the execution of specific quantitative experiments to explore the antibacterial performance of the blend material.

**Figure 13:**
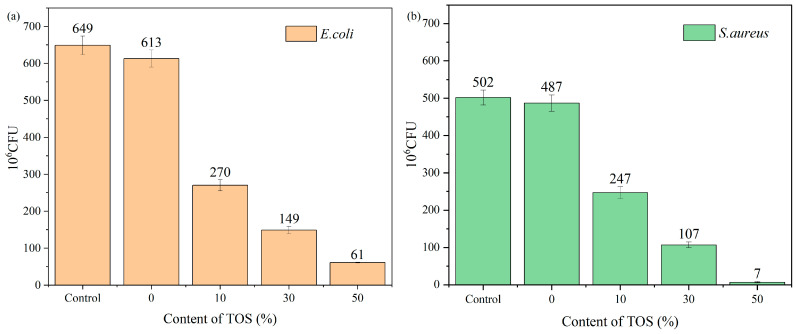
Antibacterial activity for (**a**) *E. coli* and (**b**) *S. aureus* of LDPE/TOS blends.

Figure 13a presented the results of the quantitative antibacterial tests. From the graph, it became evident that the bacterial count for pure LDPE samples was 613 × 10^6^ CFU/mL. However, the number of bacteria dramatically decreased with the addition of TOS to the blend material. When the filler content was at 10%, the bacterial count quickly fell to 270 × 10^6^ CFU/mL and continued to decrease with increasing TOS content. This confirmed that the LDPE/TOS blend exhibited a substantial inhibitory effect on the growth of *E. coli* colonies. When the filler content was at 50%, the number of bacteria in the LDPE/TOS solid medium reduced to 61 × 10^6^ CFU/mL, achieving an antibacterial inhibition rate of over 90%. Observing the antibacterial effects against *S. aureus* for all samples (Figure 13b), the trend was similar to that observed with *E. coli*, with a marked reduction in bacterial counts as the TOS content increased. However, the numbers of *S. aureus* were fewer compared to *E. coli*, indicating that TOS exerted a more pronounced inhibitory effect on *S. aureus*.

This effect can be attributed to the transformation of CaCO_3_ into CaO, which possesses antibacterial properties after heat treatment at 900 °C [13,50]. The CaO powder slurries have demonstrated bactericidal action on Gram-negative bacteria such as *E. coli*, *Salmonella typhimurium*, and *Pseudomonas aeruginosa* [51,52,53], thereby endowing LDPE with excellent antibacterial properties through the incorporation of TOS.

## 4. Conclusions

The addition of TOS, a sustainable biowaste material, to LDPE significantly enhances the properties of the resulting blend, rendering it a suitable candidate for food packaging applications. This blend demonstrated increased water resistance, mechanical strength, and antibacterial activity, thereby providing a promising solution for the burgeoning packaging market that aligns with the principles of sustainability. Specifically, the usage of this LDPE/TOS blend could extend the shelf-life of perishable food items like vegetables and chicken meat, addressing both food safety and public health concerns.

## Figures and Tables

**Table 1:** Proportion of blends of LDPE and LDPE/TOS.

Sample	LDPE (%)	TOS (%)
LDPE	100	0
LDPE/TOS (10%)	90	10
LDPE/TOS (20%)	80	20
LDPE/TOS (30%)	70	30
LDPE/TOS (40%)	60	40
LDPE/TOS (50%)	50	50
LDPE/TOS (60%)	40	60

LDPE: low-density polyethylene. TOS: thermally treated oyster shell powder.

**Table 2:** Thermogravimetric/differential analysis of LDPE/TOS blends.

Sample	*T_onset_* (°C)	*T_max_* (°C)
LDPE	259.41	500.48
LDPE/TOS (20%)	413.18	503.23
LDPE/TOS (30%)	404.35	504.47
LDPE/TOS (40%)	412.95	504.85
LDPE/TOS (50%)	418.54	505.62
LDPE/TOS (60%)	451.15	509.77

*T_onset_*: Initial degradation temperature. *T_max_*: temperature at maximum weight loss.

**Table 3:** Differential scanning calorimetry analysis of LDPE/TOS blends.

Sample	*T_g_* (°C)	*T_c_* (°C)	Δ*H_m_* (J/g)	*X_c_* (%)	*T_m_* (°C)
LDPE	69.7	96.1	−54.77	18.69	113.8
LDPE/TOS(50%)	69.9	95.8	−28.32	19.33	114.3

*T_g_*: glass transition temperature. *T_c_*: crystallization temperature. Δ*H_m_*: melting enthalpy. *X_c_*: crystallinity. *T_m_*: melting temperature.

## Data Availability

The data that support the findings of this study are available from the corresponding author, upon reasonable request.

## References

[1] Babaremu K., Oladijo O.P., Akinlabi E. (2023). Biopolymers: A suitable replacement for plastics in product packaging. Adv. Ind. Eng. Polym. Res..

[2] Agarwal A., Shaida B., Rastogi M., Singh N.B. (2023). Food packaging materials with special reference to biopolymers-properties and applications. Chem. Afr..

[3] Jordan J.L., Casem D.T., Bradley J.M., Dwivedi A.K., Brown E.N., Jordan C.W. (2016). Mechanical Properties of Low Density Polyethylene. J. Dynamic Behavior Mater..

[4] Song J.H., Murphy R.J., Narayan R., Davies G.B.H. (2009). Biodegradable and compostable alternatives to conventional plastics. Philos. Trans. R. Soc. B.

[5] Reesha K.V., Panda S.K., Bindu J., Varghese T.O. (2015). Development and characterization of an LDPE/chitosan composite antimicrobial film for chilled fish storage. Int. J. Biol. Macromol..

[6] Santos X., Rodríguez J., Guillén F., Pozuelo J., Molina-Guijarro J.M., Videira-Quintela D., Martín O. (2023). Capability of Copper Hydroxy Nitrate (Cu_2_(OH)_3_NO_3_) as an Additive to Develop Antibacterial Polymer Contact Surfaces: Potential for Food Packaging Applications. Polymers.

[7] Castillo P., Goñi-Ciaurriz L., Olate-Moya F., Bastías R., Farias S., Palza H. (2023). Polyethylene with MoS_2_ nanoparticles toward antibacterial active packaging. J. Appl. Polym. Sci..

[8] Basiron N., Sreekantan S., Akil H.M., Saharudin K.A., Harun N.H., Mydin R.B.S., Seeni A., Rahman N.R.A., Adam F., Iqbal A. (2019). Effect of Li-TiO_2_ nanoparticles incorporation in ldpe polymer nanocomposites for biocidal activity. Nano-Struct. Nano-Objects..

[9] Li D., Ye Q., Jiang L., Luo Z. (2017). Effects of nano-TiO_2_-LDPE packaging on postharvest quality and antioxidant capacity of strawberry (Fragaria ananassa Duch.) stored at refrigeration temperature. J. Sci. Food Agric..

[10] Luo Z., Wang Y., Jiang L., Xu X. (2015). Effect of nano-CaCO_3_-LDPE packaging on quality and browning of fresh-cut yam. LWT—Food Sci. Technol..

[11] Shemesh R., Krepker M., Goldman D., Danin-Poleg Y., Kashi Y., Nitzan N., Anita V., Segal E. (2015). Antibacterial and antifungal LDPE films for active packaging. Polym. Adv. Technol..

[12] Bruna J.E., Peñaloza A., Guarda A., Rodríguez F., Galotto M.J. (2012). Development of MtCu_2_^+^/LDPE nanocomposites with antimicrobial activity for potential use in food packaging. Appl. Clay Sci..

[13] Tsou C.H., Wu C.S., Hung W.S., De Guzman M.R., Gao C., Wang R.Y., Peng M.C., Suen M.C. (2019). Rendering polypropylene biocomposites antibacterial through modification with oyster shell powder. Polymer.

[14] Tsou C.H., Zeng R., Wan N., De Guzman M.R., Hu X.F., Yang T., Gao C., Wei X., Yi J., Lan L. (2023). Biological oyster shell waste enhances polyphenylene sulfide composites and endows them with antibacterial properties. Chin. J. Chem. Eng..

[15] Feng J., Li Z., Olah A., Baer E. (2018). High oxygen barrier multilayer EVOH/LDPE film/foam. J. Appl. Polym. Sci..

[16] Lagaron J.M., Catalá R., Gavara R. (2004). Structural characteristics defining high barrier properties in polymeric materials. Mater. Sci. Technol..

[17] Puebla K., Arcaute K., Quintana R., Wicker R.B. (2012). Effects of environmental conditions, aging, and build orientations on the mechanical properties of ASTM type I specimens manufactured via stereolithography. Rapid Prototyp. J..

[18] Deng R., Liu Y., Ding Y., Xie P., Luo L., Yang W. (2009). Melt electrospinning of low-density polyethylene having a low-melt flow index. J. Appl. Polym. Sci..

[19] Ben-Dor E., Banin A. (1989). Determination of organic matter content in arid-zone soils using a simple “loss-on-ignition” method. Commun. Soil Sci. Plant Anal..

[20] Vasques Mendonça A.R., Guelli U. (2015). de Souza, S.M.; Valle, J.A.; de Souza, A.A.U. Thermogravimetric analysis and kinetic study of pyrolysis and combustion of residual textile sludge. J. Therm. Anal. Calorim..

[21] Ong P.J., Leow Y., Soo X.Y.D., Chua M.H., Ni X., Suwardi A., Tan C.K.I., Zheng R., Wei F., Xu J. (2023). Valorization of Spent coffee Grounds: A sustainable resource for Bio-based phase change materials for thermal energy storage. Waste Manage..

[22] Peta K., Bartkowiak T., Galek P., Mendak M. (2021). Contact angle analysis of surface topographies created by electric discharge machining. Tribol. Int..

[23] Azmin S.N.H.M., Nor M.S.M. (2020). Development and characterization of food packaging bioplastic film from cocoa pod husk cellulose incorporated with sugarcane bagasse fibre. J. Bioresour. Bioprod..

[24] Huang J., Tong X., Yang J., Wang Z., Zhang M., Wang X., Yang J. (2020). Synthesis of poly (hexamethylene terephthalamide)-co-polycaprolactam/modified montmorillonite nanocomposites with enhanced mechanical properties and lower water absorption rate by in-situ polymerization. J. Polym. Res..

[25] Suwanposri A., Yukphan P., Yamada Y., Ochaikul D. (2014). Statistical optimisation of culture conditions for biocellulose production by Komagataeibacter sp. PAP1 using soya bean whey. Maejo Int. J. Sci. Technol..

[26] ASTM International (2013). Standard Test Method for Water Vapor Transmission Rate of Sheet Materials Using Dynamic Relative Humidity Measurement. https://enterprise2.astm.org/DOWNLOAD-/E398.1272742-1.pdf.

[27] Oves M., Rauf M.A., Aslam M., Qari H.A., Sonbol H., Ahmad I., Zaman G.S., Saeed M. (2022). Green synthesis of silver nanoparticles by Conocarpus Lancifolius plant extract and their antimicrobial and anticancer activities. Saudi J. Biol. Sci..

[28] Ge F.F., Wan N., Tsou C.H., Chen J.C., Wu C.S., De Guzman M.R., Zeng C.Y., Zhou L., Wang Y.T., Luo X. (2022). Thermal properties and hydrophilicity of antibacterial poly (phenylene sulfide) nanocomposites reinforced with zinc oxide-doped multiwall carbon nanotubes. J. Polym. Res..

[29] Chen S., De Guzman M.R., Tsou C.H., Li M., Suen M.C., Gao C., Tsou C.Y. (2023). Hydrophilic and absorption properties of reversible nanocomposite polyvinyl alcohol hydrogels reinforced with graphene-doped zinc oxide nanoplates for enhanced antibacterial activity. Polym J..

[30] Ojha N., Pradhan N., Singh S., Barla A., Shrivastava A., Khatua P., Rai V., Bose S. (2017). Evaluation of HDPE and LDPE degradation by fungus, implemented by statistical optimization. Sci. Rep..

[31] Tjong S.C., Liang G.D. (2000). Microstructural characteristics and tensile properties of polypropylene composites filled with silver nanoplatelets. Polymer.

[32] Premalal H.G.B., Ismail H., Baharin A. (2002). Comparison of the mechanical properties and interfacial interactions between talc, kaolin, and calcium carbonate filled polypropylene composites. J. Appl. Polym. Sci..

[33] Tsou C.H., Du J.H., Yao W.H., Fu L., Wu C.S., Huang Y., Qu C.L., Liao B. (2023). Improving Mechanical and Barrier Properties of Antibacterial Poly (Phenylene Sulfide) Nanocomposites Reinforced with Nano Zinc Oxide-Decorated Graphene. Polymers.

[34] AVCIOĞLU S. (2022). LDPE matrix composites reinforced with dysprosium-boron containing compounds for radiation shielding applications. J. Alloys Compd..

[35] Prabhakar M.N., Shah A.U.R., Rao K.C., Song J.I. (2015). Mechanical and thermal properties of epoxy composites reinforced with waste peanut shell powder as a bio-filler. Fibers Polym..

[36] Wang K., Liang S., Deng J., Yang H., Zhang Q., Fu Q., Dong X., Wang D., Han C.C. (2006). The role of clay network on macromolecular chain mobility and relaxation in isotactic polypropylene/organoclay nanocomposites. Polymer.

[37] Naz A., Riaz I., Jalil R., Afzal S. (2021). Creep strain and recovery analysis of polypropylene composites filled with graphene nano filler. Polymer.

[38] Zeng D., Su Y., Dong J., Ma X., Yu L., Lv Y. (2022). Investigation on improvement of thermal, mechanical, and barrier properties of lignin/poly (3-hydroxybutyrate-co-3-hydroxyhexanoate) using inorganic nanoparticles as fillers. Polym. Compos..

[39] Chrissafis K., Bikiaris D. (2011). Can nanoparticles really enhance thermal stability of polymers? Part I: An overview on thermal decomposition of addition polymers. Thermochimica Acta..

[40] Bello A.M., Haladu I. (2023). A Guinea Fowl Eggshells Derived Calcium Oxide Catalyst for Transesterification of Coconut Oil. Univers. J. Catal. Sci..

[41] Yang S.J., Song W.J., Dingwell D.B., He J., Guo H.B. (2022). Surface roughness affects metastable non-wetting behavior of silicate melts on thermal barrier coatings. Rare Met..

[42] Tsou C.H., Ma Z.L., De Guzman M.R., Zhao L., Du J., Emori W., Gao C., Zhao Y., Yang T., Wu J. (2022). High-performance antibacterial nanocomposite films with a 3D network structure prepared from carboxylated graphene and modified polyvinyl alcohol. Prog. Org. Coat..

[43] Kumar S.S.A., Bashir S., Ramesh K., Ramesh S. (2021). New perspectives on Graphene/Graphene oxide based polymer nanocomposites for corrosion applications: The relevance of the Graphene/Polymer barrier coatings. Prog. Org. Coat..

[44] Tsou C.H., Ge F.F., Lin L., Yuan S., De Guzman M.R., Potiyaraj P. (2023). Barrier and Biodegradable Properties of Poly (butylene adipate-co-terephthalate) Reinforced with ZnO-Decorated Graphene Rendering it Antibacterial. ACS Appl. Polym. Mater..

[45] Griffin A., Guo Y., Hu Z., Zhang J., Chen Y., Qiang Z. (2022). Scalable methods for directional assembly of fillers in polymer composites: Creating pathways for improving material properties. Polym. Compos..

[46] Zia J., Paul U.C., Heredia-Guerrero J.A., Athanassiou A., Fragouli D. (2019). Low-density polyethylene/curcumin melt extruded composites with enhanced water vapor barrier and antioxidant properties for active food packaging. Polymer.

[47] Zhou Y., Zhang L., Zheng M., Chen F. (2017). Improved Water Resistance and Antibacterial Activity of a Novel Chitosan–Glucose–Stearic Acid Composite. ACS Appl. Mater. Interfaces..

[48] Zhang J., Tan W., Han Y., Dong Z. (2019). Mechanism of LDPE/wood flour composites with two sizes of wood flour. Polymers.

[49] Bolton D.J., Meredith H., Walsh D., McDowell D.A. (2014). The effect of chemical treatments in laboratory and broiler plant studies on the microbial status and shelf-life of poultry. Food Control.

[50] Tongwanichniyom S., Kitjaruwankul S., Phornphisutthimas S. (2022). Production of biomaterials from seafood waste for application as vegetable wash disinfectant. Heliyon.

[51] Sawai J., Igarashi H., Hashimoto A., Kokugan T., Shimizu M. (1995). Evaluation of growth inhibitory effect of ceramics powder slurry on bacteria by conductance method. J. Chem. Eng. Jpn..

[52] Sawai J., Kojima H., Igarashi H., Hashimoto A., Shoji S., Takahara A., Sawaki T., Kokugan T., Shimizu M. (1997). Escherichia coli damaged by ceramic power slurry. J. Chem. Eng. Jpn..

[53] Roy A., Gauri S.S., Bhattacharya M., Bhattacharya J. (2013). Antimicrobial activity of CaO nanoparticles. J. Biomed. Nanotechnol..

